# Complex IoT Systems as Enablers for Smart Homes in a Smart City Vision

**DOI:** 10.3390/s16111840

**Published:** 2016-11-02

**Authors:** Per Lynggaard, Knud Erik Skouby

**Affiliations:** Center for Communication and Information Technologies Aalborg University Denmark; A. C. Meyers Vænge 15, 2450 Copenhagen SV, Denmark; skouby@es.aau.dk

**Keywords:** IoT, cloud-of-things (CoT), smart city, smart home, wireless sensor network (WSN), energy harvesting

## Abstract

The world is entering a new era, where Internet-of-Things (IoT), smart homes, and smart cities will play an important role in meeting the so-called big challenges. In the near future, it is foreseen that the majority of the world’s population will live their lives in smart homes and in smart cities. To deal with these challenges, to support a sustainable urban development, and to improve the quality of life for citizens, a multi-disciplinary approach is needed. It seems evident, however, that a new, advanced Information and Communications Technology ICT infrastructure is a key feature to realize the “smart” vision. This paper proposes a specific solution in the form of a hierarchical layered ICT based infrastructure that handles ICT issues related to the “big challenges” and seamlessly integrates IoT, smart homes, and smart city structures into one coherent unit. To exemplify benefits of this infrastructure, a complex IoT system has been deployed, simulated and elaborated. This simulation deals with wastewater energy harvesting from smart buildings located in a smart city context. From the simulations, it has been found that the proposed infrastructure is able to harvest between 50% and 75% of the wastewater energy in a smart residential building. By letting the smart city infrastructure coordinate and control the harvest time and duration, it is possible to achieve considerable energy savings in the smart homes, and it is possible to reduce the peak-load for district heating plants.

## 1. Introduction

Visions of future smart cities have been presented by many countries [1] and institutions [2]. The common element in these visions for smart cities [3] is to move the urban development in a direction that improves the quality of life for the citizens in the form of enhancing livability, sustainability, and efficiency [4]. Thus, smart cities manage and integrate the city’s assets such as urban mobility, energy management, waste management, water management, and e-governance into a united global concept [5].

To realize this vision, a digital infrastructure must be provided. This infrastructure must be based on a modern Information and Communications Technology ICT solution that is able to combine and integrate services and technologies into one united and universal platform. The key technology drivers for this vision comprise 5G communication, Cloud-of-Things (CoT), and Internet-of-Things (IoT) [6]. Hence, Gartner expects that 20.8 billion IoTs are connected to the internet by 2020 [7].

This paper presents and elaborates a vision for a smart city ICT based infrastructure that is divided into layers. These layers provide means for interfacing and integrating the individual key technologies into one united service framework. In particular, the layers that deal with the complex IoT devices in the context of smart homes and CoT technologies are in focus.

The presented model comprises four layers which encapsulate IoT, smart homes, CoT, and smart city technologies and services. The IoT layer includes the complex IoT devices, which create groups by interfacing with each other and with the smart home Artificial Intelligence AI system. At the smart home level, the smart homes are integrated and combined into CoT clusters which constitute the main smart city backbone. Similarly, the CoT and the smart city layers encapsulate the smart home, their technologies, and their services.

The main benefit of dividing these technologies into different layers is that different types of services can be deployed, i.e., the services can be specialized, encapsulated, and decoupled from each other [8]. An example could be a combined service which uses some of the IoT devices located in a smart home context. The service is handled in this context only, i.e., security, performance, and usability can be optimized locally without taking the content, services and technology of the other layers into consideration. However, to be able to integrate services between the layers, some well-defined generalized interfaces must be defined and developed. Such interfaces support a structure where composite and complex services can be handled by the higher levels. To exemplify these concrete benefits from a “real world perspective”, a selected complex IoT example is presented and discussed in detail. Hence, the pros and cons of deploying this system in the context of the presented infrastructure are explored by using a simulation model that copes with energy harvesting from wastewater in a residential building. The outcome from this simulation model clearly demonstrates that layering and grouping technologies such as complex IoTs, smart homes, and CoTs provide considerable benefits.

To sum up, this paper presents a smart city infrastructure model which is elaborated on and discussed; however, it is not possible to fully cover all aspects of the complex model in a single paper, and we have selected demonstration of parts of its usability and benefits by deploying a wastewater energy harvesting system in a smart building and letting the infrastructure model control the parameters related to the savings. By modeling and simulating this energy harvesting system, it has been found that considerable energy amounts can be saved, which indirectly means that costly peak loads on district heating plants can be reduced.

## 2. Complex IoT Systems for Smart Homes

The IoT technology is expected to integrate the Internet as we know it today into a multitude of things, and hence commonly known objects such as clothes, food packaging, toothbrushes, etc. will be equipped with some level of Internet-addressable AI [9,10]. Thus, these IoTs will offer context awareness and communication features [11], and they will share some level of pseudo-intelligence depending on their processing capability and consumed power limitation [12].

This development will lead to new forms of communication between people and things and between things themselves. Thus, the challenge is to go beyond today’s state-of-the-art, making these IoTs context-aware, intelligent and able to communicate wirelessly, and combining them into a distributed system for the future smart homes and smart cities, [13]. They should be able to not only react to changes in the environment, but also perform AI-based reasoning to take into account the preferences of the user inhabiting the smart home [14].

One of the main drivers in this forecast is the huge worldwide focus on energy savings in a global context to reach the goal of lowering the emitted carbon footprint and the greenhouse gases [15]. Thus, it is expected that future smart home devices will be equipped with IoT devices, which are able to control and limit these emissions [16], i.e., these devices are able to optimize their behavior dynamically to achieve a higher level goal. Smart homes utilize this dynamical behavior and add a high level control facility in the form of an advanced artificial intelligence framework, which communicates with other similar systems to reach a united goal for all the units; however, a lot of research is needed in this area [15].

## 3. Related Work

A proposal for a smart city architecture is presented in a paper from Balakrishna et al. [17]. They suggest a matrix structure where smart-city-areas such as smart environments, smart people, and smart living are linked together by service frameworks such as large-scale instrumentation-pervasive sensors, ubiquitous high-speed network infrastructures, and antonymous data management systems. This system architecture has the benefits of a coherent interface between all the smart-city-areas, but it lacks the interface between the different services. The lack of interfaces means that this system does not scale well with an increasing number of services. Similarly, if a new smart-city-area is added, it means that all the services must be updated to interface with this. The system presented here is able to cope with these limits because of the hierarchical architecture proposed. Thus, adding a new service or a new smart-city-area is simply performed by adding new functionality to the respective layer where the service belongs. In addition, this new functionality in a layer can be used in the upper layers to offers a more complex service. This way, the scalability problem in the matrix architecture where all sensors need to interface with each other is solved in the hierarchical architecture where the sensors are grouped locally at a layer, and the higher layers take care of communication between these groups.

A paper by Welbourne et al. [18] deals with creating a microcosm for the IoTs, which enables common devices such as washing and dishwashing machines to be able to interconnect and communicate with its environments. This technology fits very well into the smart city infrastructure model presented in this paper.

A model that handles mapping from physical things at the lowest level to user interactions at the highest level is presented by Yao et al. [10]. Their eight-layer model fits into the layers of the model presented in this paper. The first layers of the Yao et al. model collect events from physical things, process them in an event manager, and store them in a database that offers user access. These layers can easily be mapped into layer 1 and layer 2 of the presented model, where layer 1 handles the “physical things” and the “event manager” parts, whereas layer 2 handles the “database” and the “user interface” parts.

A paper from Ghayvat et al. [16] presents a framework that is able to assist the elderly to stay longer at home, i.e., Ambient Assisted Living, which is one of the important issues in the big challenges [19]. The architecture of their system is deploying a ZigBee network where the nodes are grouped into the respective rooms in the instrumented home, and the data processing and the collecting unit are allocated on a dedicated smart home server. This solution offers the possibility to connect a cloud-based server that integrates with the healthcare providers. The system fits very well into the layered framework presented in this paper. Thus, the ZigBee nodes are grouped into the lowest layer, which is connected into the smart home server positioned at the second layer. Finally, this server is integrated with the CoT server in the third layer. A similar work has been performed in [20].

The work performed by Nasir et al. [21] discusses a cognitive network for monitoring and predicts the water usage in smart homes. They mount Radio Frequency Identification RFID sensors at the water pipes and process the data from these in a hidden Markov model to predict events, identify patterns, and making decisions. By using their framework in combination with the wastewater energy recovery system discussed in this paper, a fully automated system can be derived which is adaptive and able to predict optimal usage of the water resources, e.g., when to take a shower and when to run the washing and dishwasher machines.

## 4. A Vision for a Smart City Infrastructure

A smart city vision must deal with the multitude of challenges of a smart city context as discussed earlier. To be able to organize these challenges in a structured manner, a hierarchical Smart City Infrastructure (SCI) model is needed. This paper presents such a SCI model which is related to work performed by the authors in [22].

**Figure 1:**
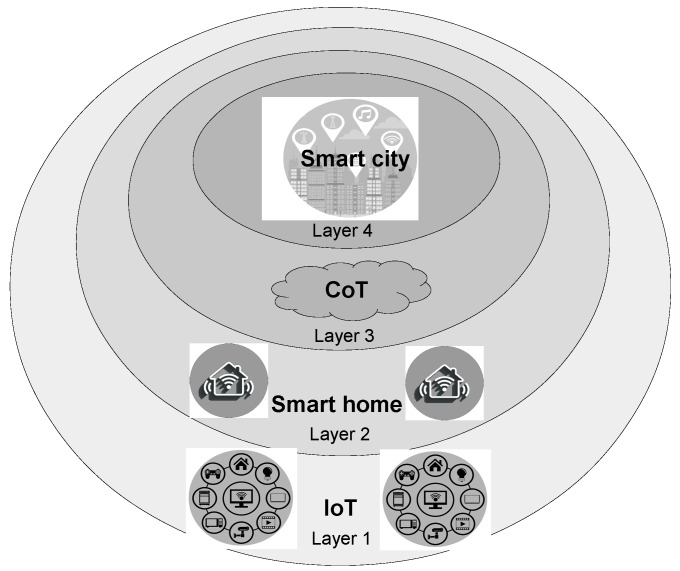
Information and Communications Technology ICT based infrastructure for future smart cities illustrated as a hierarchical model.

An SCI model for future smart cities is illustrated in Figure 1 as a hierarchical model [22]. The first layer (IoT) comprises a collection of complex IoTs, which offer context awareness, communication capability, and artificial intelligence. The second layer (smart home) shows the smart homes that embed the complex IoTs and interface these to a local smart home server, which offers local services, advanced AI computation, and interface possibilities for the users. The third layer (CoT) integrates the smart home AI systems into CoT services that are able to combine information from the individual smart homes and thereby offer more composite smart city services. Layer 4 provides additional service infrastructures such as smart grids for resource distribution, intelligent transportation systems, social services, and integration with 5G machine-to-machine communication, etc.

The SCI offers services and advantages in areas such as “environment, energy and water”, “government, administration and public safety”, and “social programs and healthcare”.

Firstly, in the area “environment, energy and water”, the SCI offers automated handling of resource scheduling by using the smart home IoT and AI systems in combination with the CoT system. This combination offers a backbone which is able to manage resources by coordinating them. An example is coordinating the distribution of electricity to the consumers, which improves the efficiency, reliability, economics, and sustainability of its production and distribution. Similarly, using complex IoT systems to monitor sustainable resources like rainwater, solar based heating, wastewater, and power production systems can provide local and global savings by combining these systems into a shared resource pool which the SCI coordinate and control.

Secondly, using the SCI system in the area “government, administration and public safety” allows for advanced taxation services. These services can be directly linked to the resource consumption and pollution costs by using complex IoT systems in combination with a SCI. In addition, the smart home AI part can watch for abnormal behavior and alarm neighbors, police, security companies, etc. if this is needed.

Lastly, the area “social programs and healthcare” offers a multitude of services such as: common information server (CoT-based), which partly updates automatically based on the smart home AI observations; AI organizes and recommends common shopping; AI can schedule activities such as looking after children, pets, etc.; AI is able to recommend social relationships (like LinkedIn); AI detects unusual behavior like an elderly person has fallen; and, within telemedicine, AI supervises behavior and informs caregivers, calls emergency teams, etc.

The suggested SCI has some challenges related to security, privacy, costs, usability, user involvement, and the fact that the advanced AI does not exist. This is a major challenge that has to be addressed and overcome if the benefits of advanced ICT systems and data coordination shall be realized. The first steps to address these challenges are taken with the European Union General Data Protection Regulation (EU GDPR)-directive [23], but a lot still needs to be done in this area, as free, but controlled flows of data are required in the smart infrastructure.

Another major challenge in the SCI model is to integrate the complex IoT system with the smart homes and the smart city ICT [24]. A complex IoT system that comprises state-of-the-art wireless sensor communication, artificial intelligence, and sensing capabilities has challenges in form of network bandwidth, quality of service expectations, and the ability to handle the huge amount of produced sensor-information (i.e., big-data). In particular, the AI-part is challenging because it is embedded into the IoT context, which offers limited processing ability, limited power resources, and limited bandwidth resources [24]. Thus, an advanced AI system is needed for handling the complex IoT patterns in the smart home context as well as in the smart city context. This AI system could be implemented as a distributed system where one part is allocated in the smart homes, and the other part is allocated on the Internet as a Cloud of Things (CoT) service. Such an approach offers:

- the possibility to interconnect IoTs, coordinate activities, collect big-data, and offer complex services to the community, as well as to the individual smart home user;
- scalability—It is easy to add new smart city members in the form of smart homes, upgrade and perform service to a distributed system, i.e., it scales well;
- compatibility with the concept of smart grids enables supply of resources to a smart city.

The benefits of centralized systems such as easy access to all information and simple peripheral units can be achieved by processing the distributed pre-processed (anonymous) big-data on a cloud server.

In summary, the explored SCI is able to save resource consumption by using the resources intelligently and by coordinating their use across the smart city. Thus, it creates a cost efficient environment and better sustainability by providing a cost efficient environment for the citizens to live in, providing a high quality of life, and providing a wise management of natural resources.

To exemplify how a complex IoT system can be an enabler for a SCI vision, the section below provides a concrete example including simulations based on the ideas and the vision presented above, and considering related works (cf. next section).

## 5. Wastewater Energy Recovery in Smart Buildings—A Simulated Example

**Figure 2:**
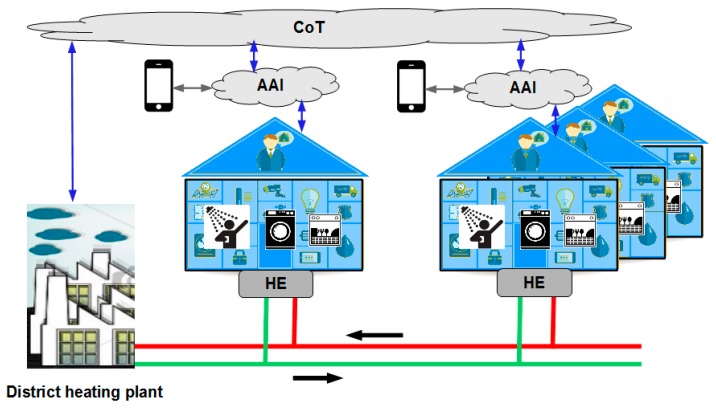
A residential building that contains two Artificial Intelligence AI equipped smart apartments.

As discussed, in the SCI model, future smart homes will be equipped with AI, which is able to control the numerous smart home devices in an intelligent manner and is able to schedule and optimize parameters for these devices [25]. To substantiate the argument, an illustrative example of such a service is presented and elaborated in the following in form of a simulation model. This model simulates the potential of harvesting energy from wastewater by coordinating and controlling the involved resources and its users. However, the simulation model is limited to the following SCI elements: smart city district heating plants; smart homes (smart buildings); IoT devices and IoT based systems in smart homes; and the CoT infrastructure needed to enable communication between the smart homes (Figure 2).

As illustrated in Figure 2, the smart homes (residential buildings) with their embedded IoT devices (a washing machine, a dishwasher, and a shower) are connected to a district heating plant that delivers hot water. When the hot water arrives at the smart home Heat Exchanger (HE), it delivers its energy and the cooled water is returned to the district heating plant. By combining two HEs, it is possible to deliver hot water to the smart homes and at the same time harvest energy from the wastewater [26]. Thus, when a smart home user takes a shower, it is possible to harvest energy from the wastewater and reuse this e.g., in another shower (connected to the same HE unit). It is noted that the scheduling of the run time for the showers can be coordinated by the users through the AI and the CoT parts in the SCI model. Similarly, by scheduling the run time of washing machines and dishwashers the wastewater energy from these can be harvested and reused e.g., for a shower taking place at the same time (in the same building). From a smart city perspective, harvesting wastewater energy means that the peak loads on the district heating plants is reduced [27], i.e., a load balancing scheme can be deployed. Reducing the variation in loads for a district heating plants has a considerable impact on its efficiency, especially at cold days where the main heat-pumps cannot deliver beyond the “base-load”, which means that backup heat plants are turned on for the price of burning costly fossil fuel or electricity [27]. This load balancing scheme is organized and controlled by the interconnected smart home AIs and the CoT interface to the district heating plants.

**Figure 3:**
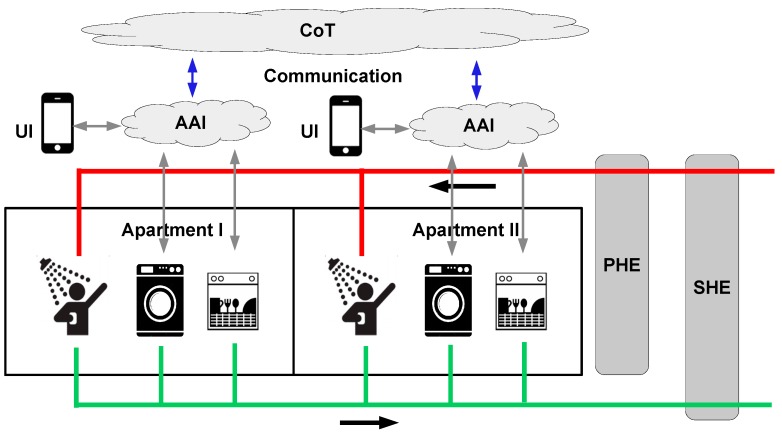
A residential building that contains two AI equipped smart apartments.

To simplify the simulations without loss of generality for the overall objective, it is assumed that two apartments in a residential building utilize the system illustrated in Figure 3, but with the deviation that the simulations focus on one smart home in the form of a residential building with a collection of apartments. This means that the results from these simulations provide the savings for some apartments in a residential building; nevertheless, from a smart city perspective, these savings can be coordinated and e.g., used for load balancing of district heating plants, as discussed earlier. It is noted that the simulated results can be generalized to include a large number of residential buildings contained in large smart cities. In this context, the residential buildings are grouped and each individual group is connected to one district heating plant (this structure already exists in many cities today). By organizing and scheduling the individual savings from each group member (each individual residential building), a load balance for the district heating plant in each individual group can be achieved. This organizing and scheduling task can be implemented as a service in layer 4 which uses layer 3 (CoT) to interconnect the group and its supplying district heating plant.

The simulated example is illustrated in Figure 3. It contains a residential building that contains two AI equipped smart apartments. The two apartments contain a shower, a washing machine, and a dishwasher, which are connected to a common drainage system. This system leads the wastewater through the Secondary Heat Exchanger (SHE), which transfers a fraction of the depleted energy from the wastewater to the hot water supply. Hot water is generated by first heating the cold water that enters the SHE with harvested energy. Next, the water leaves the SHE and enters the Primary Heat Exchanger (PHE), which is an existing component in many building today where it exchanges heat produced by a heating station. After reaching its final temperature, the hot water is supplied to the consuming apartments. It is noted that the complex IoT systems embedded in the washing machines and the dishwashers are connected to the apartment AI systems, which are able to interact with the users and schedule the activities through the smart city CoT systems. Thus, the AI system is able to schedule smart home activities, such as taking a shower, according to the user’s preferences, and, at the same time, minimizing the energy cost for the smart homes and the smart city.

### 5.1. Wastewater Energy Recovery

The produced wastewater can be categorized by its origin, i.e., the wastewater can arrive from both external and internal sources in the form of cold and hot water. External sources are water supplied from a source outside the home context, i.e., the hot water is produced by a primary heat exchanger, which is connected to an external heating system such as a centralized building heating system, a distinct heating system, or a solar heating system. Internal sources cover hot wastewater produced by machines and equipment such as washing machines, dishwashers, and cooking systems where the heat is generated from electricity.

**Figure 4:**
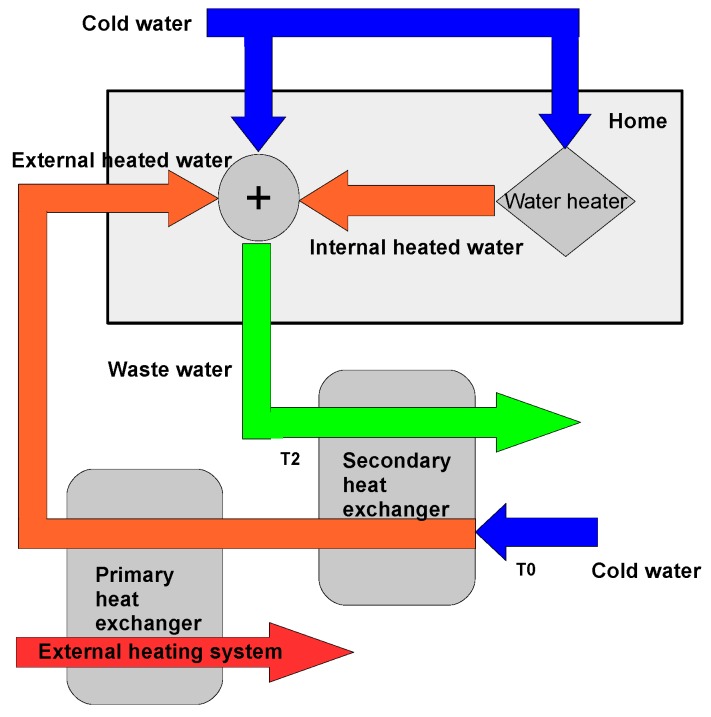
Energy harvest from internal and external wastewater sources.

To be able to harvest the energy from both the internal and external sources, a second heat exchanger can be mounted as illustrated in Figure 4.

The second heat exchanger (secondary heat exchanger, Figure 4) uses the energy in the wastewater to preheat the cold water before it arrives to the primary heat exchanger. This process saves energy in the primary heat exchanger because its differential temperature is reduced. To quantize this energy savings, it is described in a mathematical context where it is assumed that the losses from the long hot water pipes and the drainage pipes can be ignored. This assumption is justified by using insulated pipes for both the hot water and the drainage system, which is the case for most hot water pipes today. However, the drainage pipes in most buildings need to be isolated.

Regarding the heat exchanger, its performance can be expressed by using the Number of Transfer Units (NTU) effectiveness method [28,29]. This method is advantageous if only the inlet temperatures, the respective mass flow rates, and the value of the overall heath transfer coefficient are known [28]:

(1) $$NTU=\frac{UA}{C_{\text{min}}}\;\text{where}\;C_{\text{min}}=\text{min}(C_{c},C_{h})\;and\;C_{\text{max}}=\text{max}(C_{c},C_{h}),$$

where *U* (J·s^−1^·m^−2^·K^−1^) is the heat transfer coefficient of the heat exchanger, *A* (m^2^) is the total plate area inside the heat exchanger, and *C* min is the lowest heat capacitance rate at the input of either the cold water side (*C_c_*) or the hot water side (*C_h_*).

The heat exchanger effectiveness (ε) depends on the chosen type of heat exchanger. In this paper, a Plate Heat Exchanger (PHE) is used because it has a high heat transfer coefficient *U* in the range 1000–4000 [30]. The governing equation for its effectiveness is presented in (2):

(2) $$\varepsilon =\frac{1-e^{\left(-NTU(1+R)\right)}}{1+R}\;\text{where}\;R=\frac{C_{\text{min}}}{C_{\text{max}}}.$$

**Figure 5:**
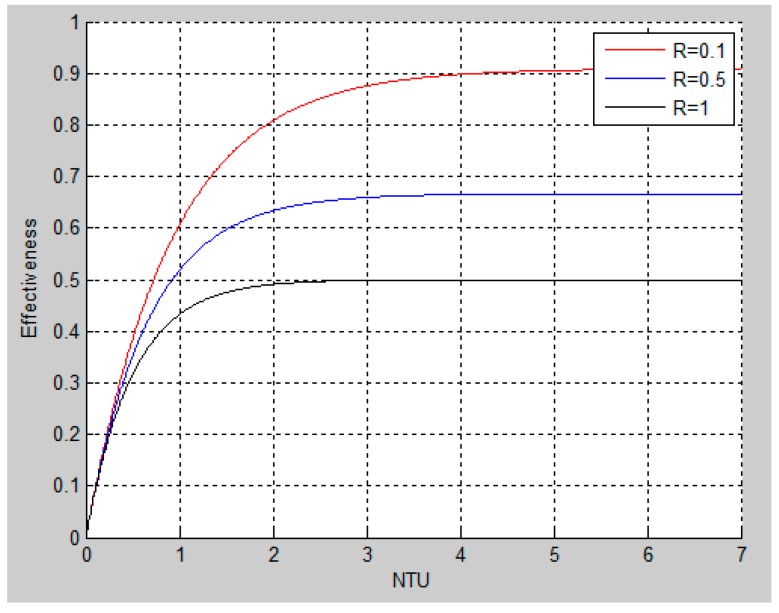
Relations between NTU and R values in a plate heat exchanger.

Plotting this function (Figure 5) gives some insight into the relation between the Number of Transfer Units NTU (1) and the *R* values.

As shown in Figure 5, a high effectiveness can be achieved by letting the flow rate R be as low as possible, and, at the same time, setting the NTU above some threshold. A low R value can be achieved by letting the hot and cold mass flow rates differ as much as possible. Similarly, a high NTU either requires a large plate area or a low mass flow rate of either the cold inlet or the hot inlet. However, in real life, the mass flow rates are given by the water usage and thereby by the produced wastewater.

### 5.2. The Simulation Model

**Figure 6:**
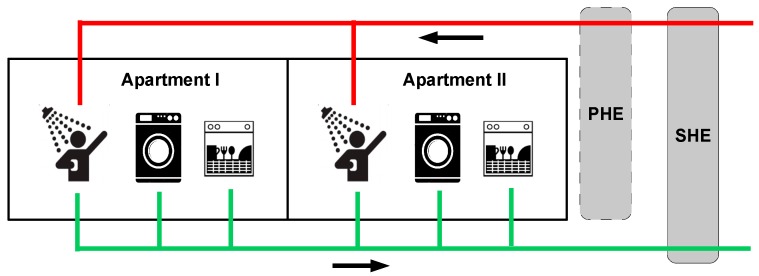
Two identical apartments equipped with IoT devices, i.e., a shower, a washing machine, and a dishwasher.

To simulate the potential for recovering energy from wastewater, a model has been developed and described in a simulation program by using the equations from the previous section. The simulation model (Figure 6) covers two identical apartments, which contain three IoT devices in the form of a shower (i.e., a user interface), a washing machine, and a dishwasher. These IoT devices are connected to an AI system that communicates, controls, and schedules their behaviors.

The energy savings in this model is achieved by controlling the runtime for each device. Hence, by adjusting the timing, it is possible to drive the Second Heat Exchanger (SHE) as close as possible to its optimal working-region or to perform load balancing for the district heating plants.

The mathematical simulation model contains two different simulations that calculate:

1 the heat exchanger efficiency as a function of simultaneous shower users;
2 the energy saved when a multiple of one dishwasher plus one washing machine run at the same time as one shower.

The fist simulation provides the amount of energy that can be saved, if the time people take a shower is coordinated. It is noted that changing people’s behavior can be achieved by presenting an economic incitement, which is related to the savings. In addition, most people care about the environment and would probably be willing to change their habits to support sustainable green energy savings. The second simulation explores the saving that can be achieved by running multiple washing machines and multiple dishwashers at the same time as a shower. It is noted that the shower and the machines do not necessarily need to be in the same apartment (but they should be in the same building to reduce heat loss in the pipes). This degree of freedom increases the likelihood that at least one dishwasher and one washing machine are ready to run when a shower takes place.

To approach real world settings, the model needs input parameters for each device such as water usage, run time, and wastewater temperature. The water usage in an apartment is subjective and depends on many factors including the usage patterns for the people living in that apartment. However, it is still possible to express the expected value (the mean) of water usage if the distribution and its parameters are known. A useable mean value of 160 L per person per day has been calculated over 27 countries in the EU. The water usage divides into the following average numbers: shower—56 L, washing machine—11 L, and dishwasher water—6 liter [31].

The run duration and wastewater temperature of a shower also need a statistical description in the form of some average values. A study performed by Wong et al. [25] which contains more than 1300 randomly picked buildings in Hong Kong provided the following average values: shower duration 12 min, shower water temperature 41 °C, and the shower wastewater temperature drop is 2–6 °C.

Similar values for the washing machine and the dishwashers are estimated to be 50 °C for the wastewater temperature and a lead-out time of 1 min. In addition, it is assumed that only half of the wastewater is heated because both dishwashers and washing machines use cold water in their initial washing phases.

The general parameters and constants used in the simulation are as follows:

- the common settings for the systems simulations;
- cold water inlet temperature (K) 7 + 273;
- water specific heat capacity (J·kg^−1^·K^−1^) 4200;
- heat transfer coefficient of the secondary heat exchanger (J·s^−1^·m^−2^·K^−1^) 1000.

### 5.3. Simulation Results

**Figure 7:**
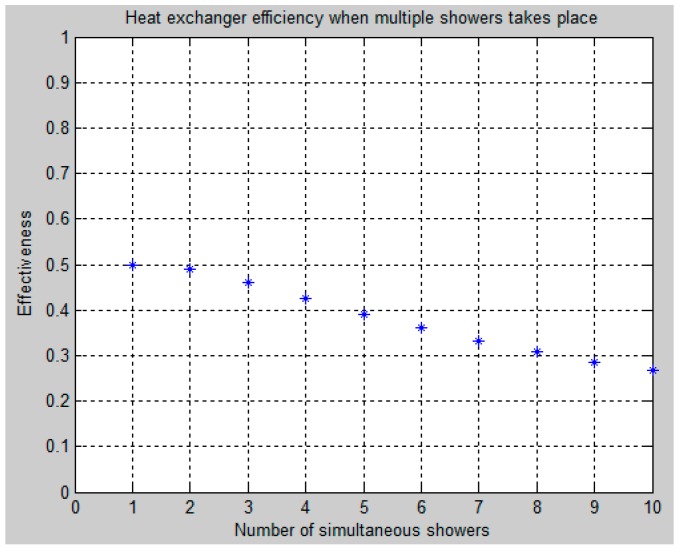
Heat exchanger effectiveness as a function of multiple simultaneous showers.

The simulation results presented in Figure 7 is the drop in heat exchanger effectiveness, if people take a shower at the same time, i.e., their wastewater are combined and fed to the Plate Heat Exchanger PHE in one single stream.

From Figure 7, it is found that taking showers one at a time sets the upper limits of the heat exchanger efficiency to 50%. However, increasing the number of simultaneous showers decreases the efficiency considerably, e.g., five simultaneous showers lower the efficiency to approximately 40% and 10 simultaneous users lower the efficiency to approximately 28%. The reason for these drops can be found in Equation (2), which describes the heat exchanger efficiency as a function of R and NTU. Even though the R factor depends on the increasing amount of hot water mass flow divided by the waste water mass flow, it is constantly one because it is assumed that the amount of wastewater equals the amount of used hot water. This assumption is justified by the fact that a shower uses the highest percentage of the consumed hot water in an apartment, as discussed previously. However, the NTU factor depends on some physical constant of the heat exchanger divided by the minimum heat capacitance rate in either the cold or hot side inlet of the heat exchanger. Thus, increasing the mass flow of water through the heat exchanger lowers its efficiency.

**Figure 8:**
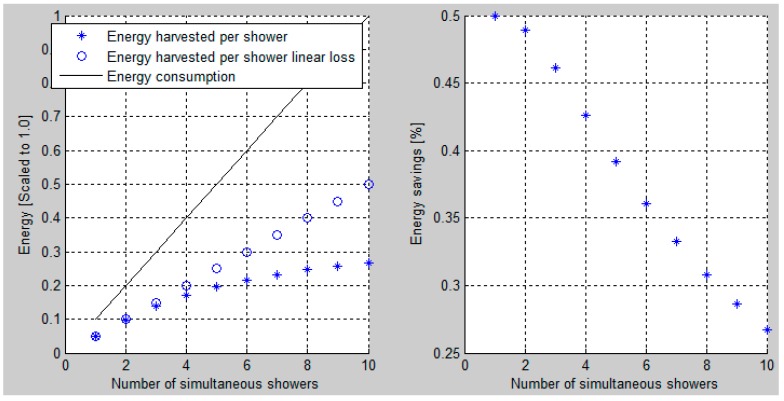
Scaled energy usage and savings in the case of multi simultaneous showers.

The calculated scaled energy consumption as a function of simultaneous showers and the previously stated assumptions is presented in Figure 8. The solid line shows the scaled total energy usage as a function of the number of simultaneous showers. Similarly, the dot-symbol-line shows the energy harvest if the heat exchanger was able to scale linearly. As expected, it is found that a linear (ideal) heat exchanger harvests approximately 50% of the energy used; however, this is not the case when deploying real world nonlinear heat exchangers where the loss depends on its load. The plus-symbol-line provides this actual harvested energy. Focusing on the saved energy, it is noted that the upper limit for the savings with one shower is approximately 50% and that it degrades down to approximately 27% when 10 showers run at the same time. Thus, scheduling the showers in residential buildings with many apartments connected to a common heat exchanger provide considerable energy savings. Thus, using a combination of AI systems and user inputs to schedule the showers dynamically provides a potential for considerable savings.

The second simulation calculates the harvested energy when a multiple number (N) of one dishwasher and one washing machine (i.e., N × (one dishwasher + one washing machine)) run at the same time as one shower. Thus, the power (J/s) for the shower is considered constant over the full shower duration and the power provided by the N × (one washing machine + one dishwasher) is harvested. The runtimes for each of these devices can be controlled and organized as stated previously.

**Figure 9:**
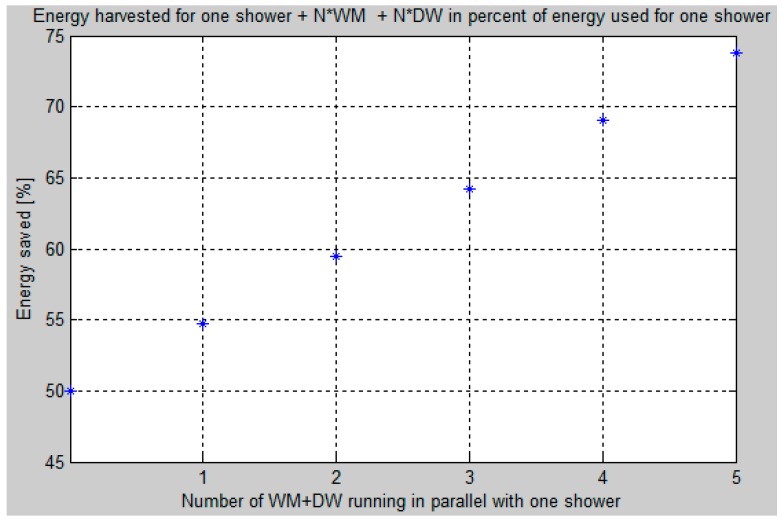
Energy harvested for one shower and N × (one washing machine + one dishwasher) in percentage of the energy used for one shower. Where N is the number of “one Dishwasher (DW) and one Washing machine (WM)” that run at the same time as one shower.

Under the condition that only one shower produces wastewater, the harvested energy will be 50% as expected (Figure 9). However, running one washing machine plus one dishwasher in parallel with the shower increases the harvest percent to 55. Similarly, the harvest percent for five washing machines plus five dishwashers increases to 74. Actually, this percentage can be “larger than 100” because the energy to the washing machine and the dishwasher comes from electricity, i.e., it does not come from the hot water supply.

## 6. Conclusions

This paper introduces a vision for a smart city model, which is inspired by the widespread visions for creating smart living in smart homes/smart cities that is addressing some of the current big societal challenges. The vision is visualized in a four-layered model providing interfaces for and integration of key technologies into a united service framework. The idea of the layered framework is to allow analytical isolation of cooperating and communicating services. As an illustration of this and to get supporting, specific data, an example is developed where IoT devices (shower, washing machine, and a dishwasher) embedded in the smart homes provide savings of energy in a smart city context, i.e., the IoT devices enable and support the discussed smart city vision. Without loss of generality, a simulation has been performed on one residential building that contains some apartments. It has been found that the simulated wastewater model deployed in the proposed smart city infrastructure is able to save from 25% to 50% of the energy if people organize their shower and between 50% to 75% if the run time of washing machines and dishwashers are organized and controlled by the distributed AI and CoT systems—A non-trivial result that indicates big potentials in the proposed smart city infrastructure model. Therefore, the details supplied in the simulation are quite elaborate in order to substantiate the claimed savings. It is noted that considerable savings are achieved locally by organizing and controlling the harvest of the wastewater energy produced by the IoT devices. These IoT devices are organized and controlled by a local AI in combination with a CoT connection, which means that it is possible to organize and control these in a global smart city context. However, the savings illustrated here are achieved locally, i.e., they reduce the transport from the district heating station and are “stand-alone” savings for each building. Similarly, global savings can be achieved by balancing the load on the district heating plants. However, the simulations do not provide evidence or data for the potential savings that can be achieved in other building-types such as single family homes, factories, and public buildings. In these building types, the hot wastewater most likely needs to be transported to an external common heat exchanger, i.e., factors like heat losses and heat exchanger efficiency must be considered. These challenges need more research and are the target for a future paper.

It is noted that similar experiments can be conducted with the electricity grids that most likely will come to the same conclusion, i.e., organizing and controlling the consumptions in an intelligent manner provides considerable savings.

Today, the technology for realizing this vision is not commercially available. It requires a lot of research and standardization activities to interconnect and interface the devices and the systems to the proposed smart city infrastructure model. At the socio-economic level, further research and political agreement are also required in a coordinated, interactive process with the technology development to achieve relevant and viable answers to the big societal challenges. An urgent example identified is cyber security and privacy where a solution is required to facilitate the data streams needed in the smart systems.
